# The health and economic burden of rare endocrine disease: Often ignored, always important

**DOI:** 10.7189/jogh.14.04249

**Published:** 2024-12-09

**Authors:** Luna Liu, Yingzhou Shi, Yuchen Li, Wanhong Wu, Yang Tian, Xiude Fan, Chao Xu

**Affiliations:** 1 Department of Endocrinology, Shandong Provincial Hospital, Shandong University, Shandong Provincial Hospital Affiliated to Shandong First Medical University, Jinan, Shandong, China; 2 Key Laboratory of Endocrine Glucose & Lipids Metabolism and Brain Aging (Shandong First Medical University), Ministry of Education, Jinan, China; 3 Shandong Clinical Research Center of Diabetes and Metabolic Diseases, Jinan, China; 4 Shandong Institute of Endocrine and Metabolic Diseases, Jinan, China; 5 Shandong Engineering Laboratory of Prevention and Control for Endocrine and Metabolic Diseases, Jinan, China; 6 Shandong Engineering Research Center of Stem Cell and Gene Therapy for Endocrine and Metabolic Diseases, Jinan, Shandong, China

## Abstract

**Background:**

Rare endocrine diseases (RED) often pose chronic and life-threatening challenges, yet their economic costs and societal impact remains have not been precisely quantified.

**Methods:**

We obtained patient data from the 2018 Nationwide Inpatient Sample (NIS) and the Nationwide Readmissions Database (NRD), identifying RED patients through International Classification of Diseases, 10th revision codes. The difference of economic burden between RED patients and the control group, including hospital length of stay, hospitalisation costs, and readmission rates, was assessed using chi-square tests.

**Results:**

We extracted 638 083 (2.98%) RED-related records from the NIS database. Compared to patients with common conditions, RED patients showed an exceedingly high burden of disease, including significantly extended hospital stays (*P* < 0.05), elevated total charges (*P* < 0.05), and heightened mortality rates (*P* < 0.05). They also had a substantially increased 30-day all-cause readmission rate based on the NRD database (*P* < 0.05). Although the different subgroups of RED patients had varying patterns of health care utilisation and economic burdens, they all surpassed those of patients with common conditions.

**Conclusions:**

There is a need for novel strategies aimed at mitigating the substantial RED-related burdens borne by individuals, families, and society in general, as well as funding for research and clinical trials.

Although each rare disease is individually uncommon, they collectively affect approximately 400 million people worldwide. This means that approximately 1 in 10 individuals struggle with one such condition, a prevalence even higher than that of type 2 diabetes [1,2]. On average, it takes roughly 4.8 years for rare disease patients to receive a correct diagnosis, and approximately 95% of rare diseases lack Food and Drug Administration (FDA) approved treatments. Recent findings from an analysis of Healthcare Cost and Utilization Project (HCUP) databases indicate that hospital discharges related to rare diseases incur significantly higher per-discharge costs and extended lengths of stay, constituting nearly half of the total national health care expenditure in the USA [3].

The existence of over 600 distinct rare diseases affecting the endocrine system has been documented by Orphanet [4], a globally renowned database dedicated to providing information on rare diseases and orphan drugs. These include inherited adrenal, pituitary, and thyroid dysfunctions, genetic disorders related to glucose and insulin homeostasis, rare anomalies in phosphocalcic metabolism, disorders impacting sexual development and maturation, and others [5]. Despite this extensive documentation, there is a lack of information regarding the prevalence, health care utilisation, and economic impact on patients with rare endocrine diseases (RED) due to the limited amount of robust epidemiological data [6]. Recent advancements in genetic diagnostics, molecular techniques, and treatment modalities, however, have been shaping the emerging therapeutic options for RED. Such data could serve as a valuable resource for informing policy implementation aimed at implementing these evolving diagnostics and therapies, as well as mitigating the substantial economic burden associated with these conditions, particularly in view of medical development for rare diseases.

We hypothesised that patients with RED experience significantly greater health care utilisation, higher economic costs, and poorer health outcomes compared to patients with common conditions. We therefore set the following research questions: ‘What is the prevalence of RED among hospitalised patients in the United States?’ and ‘How do the health care utilisation patterns (length of stay, total charges, and readmission rates) of RED patients compare to those of patients with common conditions?’

We aimed to determine the prevalence of RED among hospitalised patients using the Nationwide Inpatient Sample (NIS) and Nationwide Readmissions Database (NRD) databases; compare the length of hospital stays, total charges, and 30-day readmission rates between RED patients and those with common conditions; analyse the health care utilisation and economic burden associated with different subgroups of RED; and compare the mortality rates of RED patients with those of patients with common conditions.

## METHODS

The NIS is the largest all-payer inpatient database in the USA which provides a representative sample for assessing inpatient health care utilisation and outcomes. The NRD, meanwhile, is a nationally representative longitudinal database commonly used to evaluate readmission rates. Here we extracted data from both databases (year 2018 for NRD and 1 January 2016 to 31 December 2018 for NIS) to assess hospitalised outcomes for patients with RED in comparison to those with common conditions. RED diagnoses were determined based on the International Classification of Diseases, 10th revision (ICD-10) codes linked to these conditions or their features, primarily sourced from Orphanet. Common conditions were defined as those falling outside RED-linked codes. Our analysis encompassed 150 RED-linked diagnostic ICD-10 codes (File 2 in the **Online Supplementary Document**).

The analysis of the NIS data focussed on key hospitalised outcomes, including length of stay, total charges, and mortality rate. For the NRD database, we conducted analyses for economic costs, hospital stay, and early all-cause readmission rates within a 30-day post-initial hospitalisation period.

We presented continuous variables as means (x̄) and standard errors (SEs) and compared them using an independent two-sample *t*-test or one-way analysis of variance (ANOVA). We summarised categorical variables as frequencies and proportions and analysed for between-group differences using the χ^2^ test. We compared hospitalised outcomes, including total charges, length of stay, and readmission rates, mortality rates between patients with RED and those with common conditions. We used R, version 4.1.1 (R Core Team, Vienna, Austria) and SPSS, version 22.0 (IBM, Chicago, IL, USA) for all analyses, and considered a two-sided *P*-value <0.05 as statistically significant. We reported our findings according to STROBE guidelines [7].

## RESULTS

**Figure 1:**
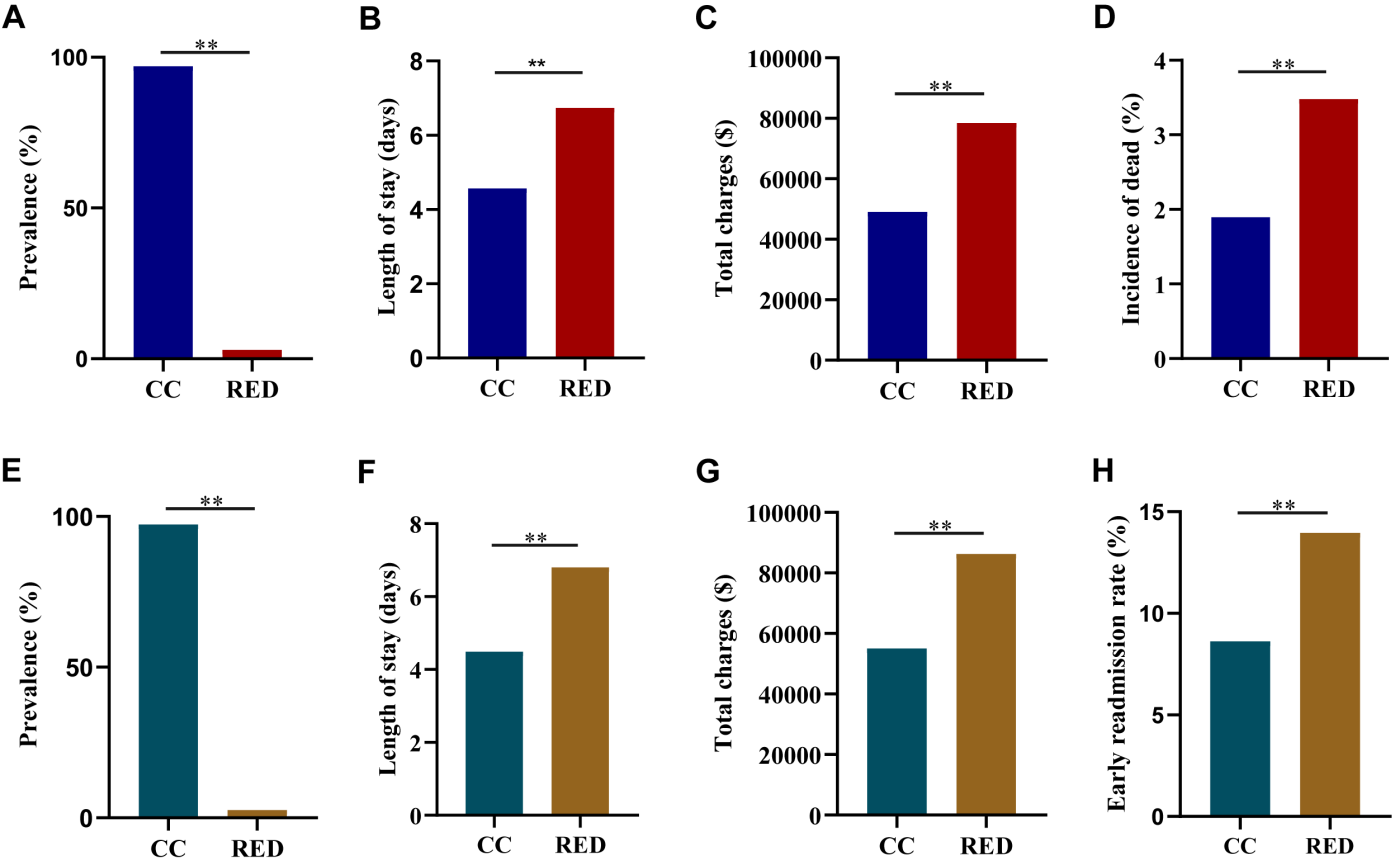
Hospitalised outcomes of inpatient with rare endocrine disease compared to common conditions based on NIS and NRD database. Continuous variables were presented as means and standard errors. **Panel A.** prevalence of RED vs. CC (NIS). **Panel B.** Length of stay in patients with RED vs. CC (NIS). **Panel C.** Total charges in patients with RED vs. CC (NIS). **Panel D.** Mortality rate in patients with RED vs. CC (NIS). **Panel E.** Prevalence of RED vs. CC (NRD). **Panel F.** Length of stay in patients with RED vs. CC (NRD). **Panel G.** Total charges in patients with RED vs. CC (NRD). **Panel H.** Early readmission rate in patients with RED vs. CC (NRD). *P < 0.01. RED – rare endocrine disease, CC – common conditions.

We identified 638 083 RED-related records in the NIS (**Figure 1**), amounting to 2.98% of the total records in the database. In comparison to patients with common conditions, those with RED had a longer length of stay (x̄ = 6.74, SE = 0.01 vs. x̄. = 4.57, SE = 0,01; *P* < 0.05) and a significant 1.60-fold increase in mean total charges (USD 78 428.30, SE = 218.40 vs. USD 49 054.51, SE = 20.57; *P* < 0.05). RED patients also had a higher risk of mortality compared to those with common conditions (3.48% vs. 1.90%; *P* < 0.05).

We additionally examined 12 928 231 patients in the NRD database and identified 344 200 (2.66%) with RED-related diagnostic codes (**Figure 1**) and found significant differences in health care utilisation between RED and common conditions. Length of stay was significantly longer for RED patients compared to common conditions (x̄ = 6.80, SE = 0.02 vs. x̄ = 4.49, SE = 0.01; *P* < 0.05). Mean total charges were approximately USD 31 270.47 higher for RED patients (USD 86 276.90, SE = 295.37) than for those with common conditions (USD 55 006.43, SE = 28.68). Based on NRD data, RED patients had a significantly higher early 30-day all-cause readmission rate than patients with common conditions (13.96% vs. 8.62%; *P* < 0.05).

**Figure 2:**
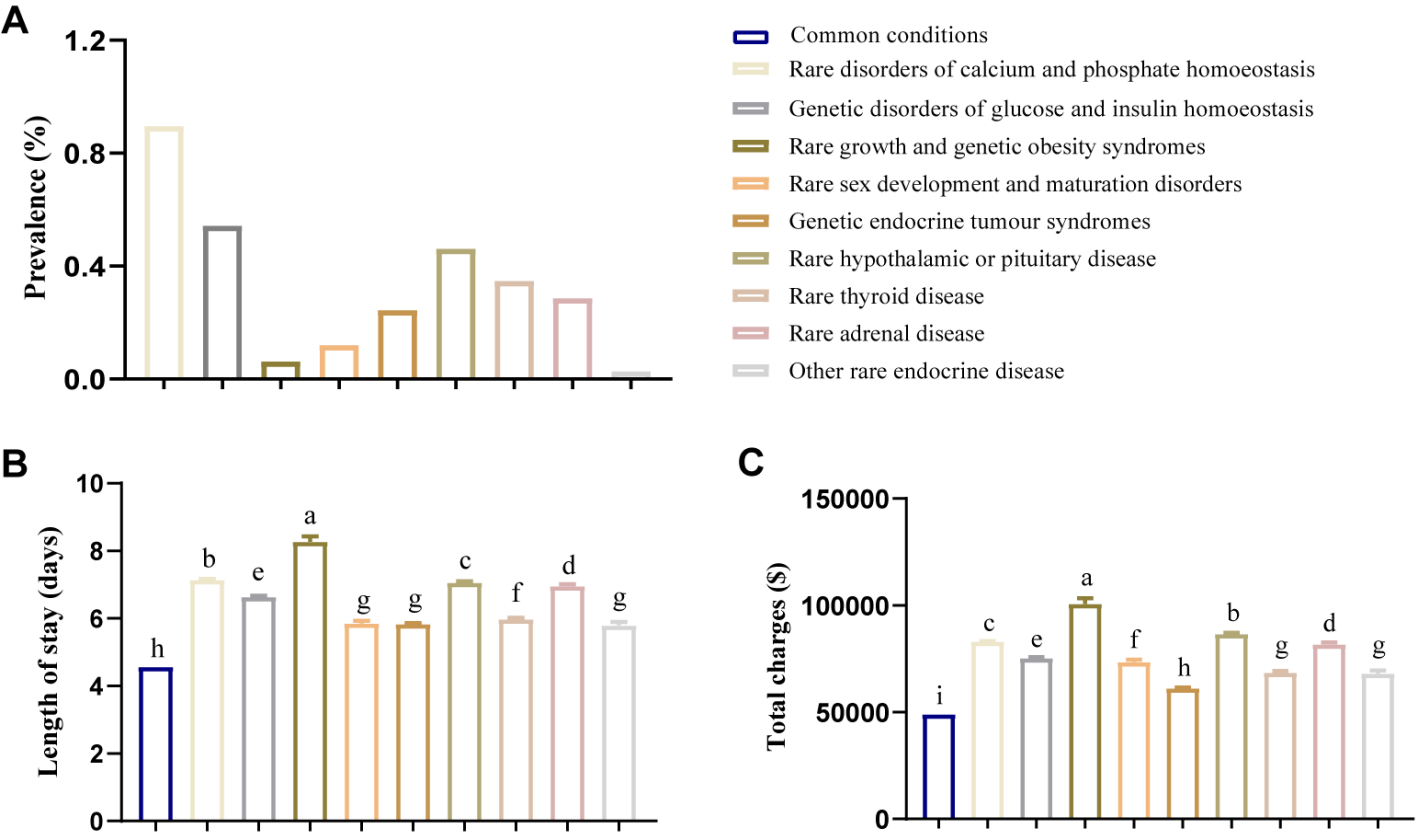
Subgroup analysis: hospitalised outcomes of inpatient with different rare endocrine disease in NIS database. Continuous variables were presented as means and standard errors. **Panel A.** The prevalence of different rare endocrine disease in whole population. **Panel B.** Length of stay in patients with different rare endocrine disease. **Panel C.** Total charges in patients with different rare endocrine disease. ABCD letter labelling for *P* < 0.05, with groups sharing at least one letter not showing significant differences and groups with different letters showing significant differences.

To further explore health care utilisation and costs for specific types of RED, we determined the percentage of different RED types and conducted in-depth analyses in both databases (**Figure 2**). In the NIS database, the most prevalent REDs were rare disorders of calcium and phosphate homeostasis (0.90%), followed by genetic disorders of glucose and insulin homeostasis (0.54%), rare hypothalamic or pituitary disease (0.46%), rare thyroid disease (0.35%), rare adrenal disease (0.29%), genetic endocrine tumour syndromes (0.24%), rare sex development disorders and maturation disorders (0.12%), rare growth and genetic obesity syndromes (0.06%), and other rare endocrine diseases (0.03%). Patients with rare growth and genetic obesity syndromes exhibited the longest hospital stays (x̄ = 8.27, SE = 0.16) and the highest total charges (USD 100 707.97, SE = 2543.37). We saw similar results in terms of prevalence for different types of RED based on the NRD database (Figure S1 in the **Online Supplementary Document**), aligning with our findings from the NIS database. However, we did find that patients with rare disorders of calcium and phosphate homeostasis possessed heaviest burden including longest length of stay (x̄ = 7.54, SE = 0.04) and highest total charges (USD 96 312.29, SE = 540.82).

## DISCUSSION

We found that individuals with RED had higher levels of health care utilisation compared to those with common conditions across both databases. This underscores the importance for policymakers, health care providers, and scientific and pharmaceutical researchers to focus on instituting supportive policies, expanding medical care, investigating underlying mechanisms, and advancing drug discovery efforts for the RED population.

REDs, often chronic and life-threatening, are significantly underrepresented in terms of their economic costs and societal impact. Based on the NIS and NRD databases, we found that between 2.66% and 2.98% of hospitalised patients were diagnosed with RED-related codes. These patients experienced significantly longer hospital stays, higher total charges, elevated mortality rates, and more frequent early all-cause readmissions compared to those with more common conditions. Furthermore, the health care utilisation and economic costs associated with subgroups of patients with different types of REDs consistently exceeded those of patients with common conditions. Addressing these issues is crucial to alleviating the societal and health care burdens faced by affected individuals and their families.

The limited epidemiological data for many rare endocrine diseases means that their true prevalence and incidence remain largely unknown. Our findings indicate that approximately 1 in 40 hospitalised patients had RED-related codes, with rare disorders of calcium and phosphate homeostasis being the most common subtype, followed by genetic disorders of glucose and insulin homeostasis. Recent research based on the HCUP databases indicates that 32% of codes corresponded to rare diseases [3], suggesting that REDs account for nearly 10% of all rare diseases, a notably high proportion. This high prevalence of calcium and phosphate homeostasis disorders may be due to their significant impact on bone metabolism [8], leading to higher rates of congenital abnormalities and disabilities, thus resulting in increased medical visits and detection.

Our results also highlight the overall societal burden of REDs, including mortality rates, hospital admission rates, and health care costs, which have been largely unexplored until now. RED patients had significantly longer length of stay, as well as higher rates of total charges, mortality rates, and early all-cause readmissions compared to patients with common conditions, with the results remaining similar in the sensitivity analyses across different RED subgroups. The mean charges for patients with RED in our study were notably higher than those for patients with rare diseases reported in previous studies based on the NIS (USD 78 428 vs. USD 69 275) and NRD (USD 86 277 vs. USD 66 675) databases [3], underscoring the importance of focussing on REDs as a distinct category. The per-person total charges for patients with RED were even higher than those for patients with lung cancer in previous studies (USD 86 277 vs. USD 74 149) [9].

The European Reference Network on Rare Endocrine Conditions survey emphasised the importance of improving functional outcomes such as social participation and workability for patients with REDs, regardless of their specific condition [10]. This underscores the need for innovative strategies and support for research and clinical trials for these conditions, which may not have previously attracted significant commercial interest. Expanding the understanding of REDs and developing targeted health care policies can significantly improve the quality of life for patients and reduce the overall economic burden on society. Future research should focus on obtaining more accurate epidemiological data and exploring the potential benefits of early diagnosis and intervention for these rare conditions. Additionally, integrating patient-reported outcomes into clinical practice could provide valuable insights into the everyday challenges faced by patients with REDs, ultimately guiding better health care strategies and policy-making.

Our study had several limitations. First, our reliance on ICD-10 codes may have lead to diagnostic inaccuracies, as these codes are not specifically validated for RED identification. Additionally, we lacked information on RED severity, relevant medications, and detailed treatment, potentially affecting the precision of our estimates. Second, we relied on data from the year 2018 and a narrow time frame (NIS data from 2016 to 2018 and NRD data from 2018), which limited our ability to capture trends and changes over time. A more extended temporal analysis would provide a more comprehensive understanding of health care utilisation and economic burden dynamics. Finally, the HCUP databases primarily capture inpatient data, excluding outpatient and primary care visits. This may not fully represent the total health care burden of RED. Future research should incorporate a broader range of data sources and longer time periods for dynamic analyses.

## CONCLUSIONS

Approximately 1 in 40 hospitalised patients in our sample had RED-related diagnostic code, with RED patients experiencing significantly higher length of stay, total charges, and early all-cause readmissions compared to common conditions patients. This highlights the urgent need for policymakers, researchers, health care providers, and employers to develop innovative measures promptly to enhance the care, management, and treatment of these debilitating diseases.

## Additional material


Online Supplementary Document
