# Supplementary material for: The health and economic burden of rare endocrine disease: Often ignored, always important
Source: J Glob Health. 2024 Dec 9;14:04249. doi: 10.7189/jogh.14.04249 (PMC11626685; doi:10.7189/jogh.14.04249)
Supplement: Online Supplementary Document [file jogh-14-04249-s001.pdf]

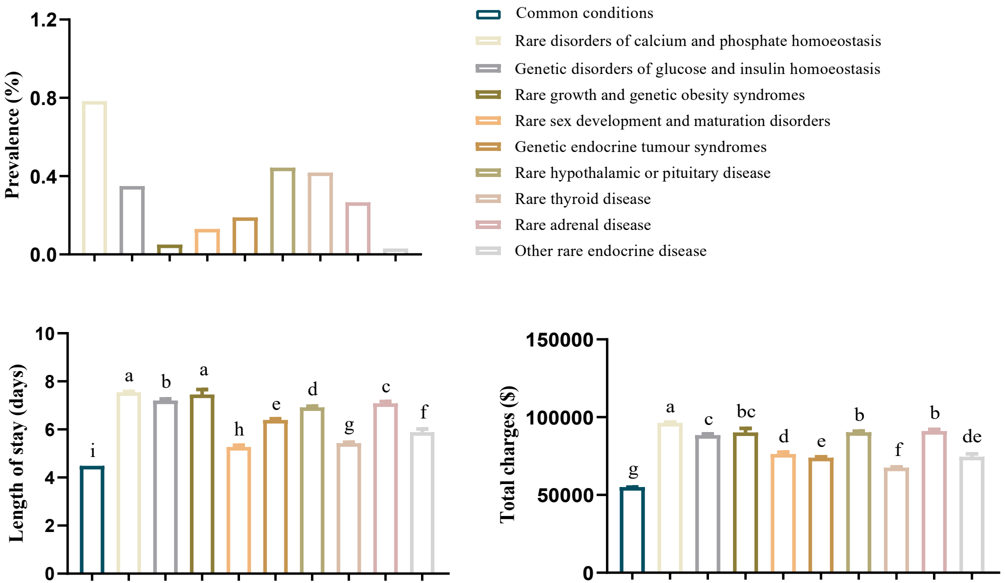

Common conditions
Rare disorders of calcium and phosphate homoeostasis
Genetic disorders of glucose and insulin homoeostasis
Rare growth and genetic obesity syndromes
Rare sex development and maturation disorders
Genetic endocrine tumour syndromes
Rare hypothalamic or pituitary disease
Rare thyroid disease
Rare adrenal disease
Other rare endocrine disease
Prevalence (%)
1.2
0.8
0.4
0.0
Length of stay (days)
10
8
6
4
2
0
i
a
b
a
h
e
d
g
c
f
Total charges ($)
150000
100000
50000
0
g
a
c
bc
d
e
b
f
b
de

Disorder	OrphaCode	Subgroup	ICD10Code
Waterhouse -Friderichsen syndrome	100067	Rare adrenal disease	'A391'
Neuroendocrine tumor of stomach	100075	Neuroendocrine neoplasm	'C169'
GRFoma	97261	Neuroendocrine neoplasm	'C259'
Germinoma of the central nervous system	91352	Rare hypothalamic or pituitary disease	'C729'
Meningioma	2495	Rare hypothalamic or pituitary disease	'D329'
Classic neuroendocrine tumor of appendix	329977	Neuroendocrine neoplasm	'D373'
Bronchial neuroendocrine tumor	97287	Neuroendocrine neoplasm	'D381'
Craniopharyngioma	54595	Rare hypothalamic or pituitary disease	'D444'
Non-functioning paraganglioma	94080	Rare adrenal disease	'D447'
Hemoglobin H disease	93616	Rare hypothalamic or pituitary disease	'D560'
Beta -thalassemia intermedia	231222	Rare hypothalamic or pituitary disease	'D561'
Pearson syndrome	699	Rare parathyroid disease and phosphocalcic metabolism anomaly	'D640'
H syndrome	168569	Rare diabetes mellitus	'D763'
Combined immunodeficiency due to LRBA deficiency	445018	Polyendocrinopathy	'D81810''D81818''D81819''D8189'
22q11.2 deletion syndrome	567	Rare parathyroid disease and phosphocalcic metabolism anomaly	'D821'
Lung fibrosis -immunodeficiency -46,XX gonadal dysgenesis syndrome	137631	Disorder of sex development	'D828'
Sarcoidosis	797	Rare hypothalamic or pituitary disease	'D860'
Proteasome -associated autoinflammatory syndrome	324977	Primary lipodystrophy	'D8982''D89831''D89832''D89833''D89834''D89835''D89839''D8989'
Congenital hypothyroidism	442	Rare thyroid disease	'E000'
Familial thyroid dyshormonogenesis	95716	Rare thyroid disease	'E030'
Thyroid ectopia	95712	Rare thyroid disease	'E031'
Obesity -colitis -hypothyroidism -cardiac hypertrophy -developmental delay syndrome	88643	Rare thyroid disease	'E038'
Rare hyperthyroidism	181399	Rare thyroid disease	'E0510''E0511'
Familial hyperthyroidism due to mutations in TSH receptor	424	Rare thyroid disease	'E0580''E0581'
IgG4 -related thyroid disease	64744	Rare thyroid disease	'E065'
Pendred syndrome	705	Rare thyroid disease	'E071'
Resistance to thyroid hormone due to a mutation in thyroid hormone receptor alpha	566231	Rare thyroid disease	'E0789'
Insulin -resistance syndrome type A	2297	Rare diabetes mellitus	E1300''E1301''E1310''E1311''E1321''E1322''E1329''E13311''E13319''E13321''E133211''E133212''E133213''E133219''E13329''E133291''E133292''E133293''E133299''E13331''E133311''E133312''E133313''E133319'<br>E13339''E133391''E133392''E133393''E133399''E13341''E133411''E133412''E133413''E133419''E13349''E133491''E133492''E133493''E133499''E13351''E133511''E133512''E133513''E133519''E133521'<br>E133522''E133523''E133529''E133531''E133532''E133533''E133539''E133541''E133542''E133543''E133549''E133551''E133552''E133553''E133559''E13359''E133591''E133592''E133593''E133599''E1336''E1337X1'<br>E1337X2''E1337X3''E1337X9''E1339''E1340''E1341''E1342''E1343''E1344''E1349''E1351''E1352''E1359''E13610''E13618''E13620''E13621''E13622''E13628''E13630''E13638''E13641''E13649''E1365''E1369''E138''E139'
Wolfram -like syndrome	411590	Rare diabetes mellitus	'E138'
Diazoxide -resistant focal hyperinsulinism due to SUR1 deficiency	276598	Hyperinsulinemic hypoglycaemia	'E161'
Zollinger - Ellison syndrome	913	Neuroendocrine neoplasm	'E164'
PPoma	97278	Neuroendocrine neoplasm	'E168'
Rare hypoparathyroidism	181405	Rare parathyroid disease and phosphocalcic metabolism anomaly	'E200'
Pseudohypoparathyroidism type 1A	79443	Rare parathyroid disease and phosphocalcic metabolism anomaly	'E201'
Familial isolated hypoparathyroidism due to agenesis of parathyroid gland	2239	Rare parathyroid disease and phosphocalcic metabolism anomaly9	'E208'
Familial isolated hyperparathyroidism	9879	Rare parathyroid disease and phosphocalcic metabolism anomaly	'E210'
Acromegaly	963	Rare hypothalamic or pituitary disease	E220'
Familial hyperprolactinemia	397685	Rare hypothalamic or pituitary disease	'E221'
Idiopathic central precocious puberty	169615	Rare endocrine growth disease	'E228'
Short stature due to isolated growth hormone deficiency with X-linked hypogammaglobulinemia	632	Rare hypothalamic or pituitary disease	'E230'
Post-traumatic pituitary deficiency	95619	Rare hypothalamic or pituitary disease	'E231'
Hereditary central diabetes insipidus	30925	Rare hypothalamic or pituitary disease	'E232'
Hypothalamic adipsic hypernatraemia syndrome	443101	Rare hypothalamic or pituitary disease	'E233'
Isolated follicle stimulating hormone deficiency	52901	Rare hypothalamic or pituitary disease	'E236'
Cushing disease	96253	Rare hypothalamic or pituitary disease	'E240'
Nelson syndrome	199244	Rare hypothalamic or pituitary disease	'E241'
Cushing syndrome due to ectopic ACTH secretion	99889	Neuroendocrine neoplasm	'E243'
Primary pigmented nodular adrenocortical disease	189439	Rare adrenal disease	'E248'
Cushing syndrome	553	Rare adrenal disease	'E249'
Classic congenital lipoid adrenal hyperplasia due to STAR deficency	325524	Disorder of sex development	'E250'
MIRAGE syndrome	494433	Rare adrenal disease	'E258'
Familial hyperaldosteronism type I	403	Rare adrenal disease	'E2609'
Apparent mineralocorticoid excess	320	Rare adrenal disease	'E261'
Ectopic aldosterone -producing tumor	231632	Rare adrenal disease	'E2681''E2689'
Familial adrenal hypoplasia with absent pituitary luteinizing hormone	95700	Rare adrenal disease	'E271'
Acute adrenal insufficiency	95409	Rare adrenal disease	'E272'
Triple A syndrome	869	Rare adrenal disease	'E2749'
Corticosteroid -binding globulin deficiency	199247	Rare adrenal disease	'E278'
Non-acquired premature ovarian failure	95710	Rare endocrine growth disease	'E2839'
Acquired premature ovarian failure	95709	Rare endocrine growth disease	'E288'
46,XY disorder of sex development due to 17-beta-hydroxysteroid dehydrogenase 3 deficiency	752	Disorder of sex development	'E291'
Familial male-limited precocious puberty	3000	Rare endocrine growth disease	'E301'
Autoimmune polyendocrinopathy type 1	3453	Rare adrenal disease	'E310'
Bangstad syndrome	1227	Polyendocrinopathy	'E318'
Carcinoid syndrome	100093	Neuroendocrine neoplasm	'E340'
Short stature due to GHSR deficiency	314811	Rare hypothalamic or pituitary disease	'E343'
Partial androgen insensitivity syndrome	90797	Disorder of sex development	'E3450''E3451''E3452'
Polyendocrine -polyneuropathy syndrome	453533	Rare thyroid disease	'E348'
Hypocalcemic vitamin D-dependent rickets	289157	Rare parathyroid disease and phosphocalcic metabolism anomaly	'E550'
Obesity due to congenital leptin deficiency	66628	Rare hypothalamic or pituitary disease	'E668'
X-linked cerebral adrenoleukodystrophy	139396	Rare adrenal disease	'E7130''E71310''E71311''E71312''E71313''E71314''E71318''E7132''E7139'
Hyperinsulinism -hyperammonemia syndrome	35878	Hyperinsulinemic hypoglycaemia	'E728''E7281''E7289'
Classic galactosemia	79239	Rare endocrine growth disease	'E7420''E7421''E7429'
Chronic visceral acid sphingomyelinase deficiency	77293	Rare dyslipidemia	'E7521''E7522''E7523''E75240''E75241''E75242''E75243''E75248''E75249''E7525''E7526''E7529'
Wolman disease	75233	Rare dyslipidemia	'E755'
TMEM199 -CDG	466703	Rare dyslipidemia	'E778'
Hypercholesterolemia due to cholesterol 7alpha-hydroxylase deficiency	209902	Rare dyslipidemia	'E7801'
Cholesterol -ester transfer protein deficiency	79506	Rare dyslipidemia	'E784'
Fish -eye disease	79292	Rare dyslipidemia	'E786'
X-linked hypophosphatemia	89936	Rare parathyroid disease and phosphocalcic metabolism anomaly	'E8331''E8332'
Familial hypocalciuric hypercalcemia type 1	93372	Rare parathyroid disease and phosphocalcic metabolism anomaly	'E8350''E8359'
Familial partial lipodystrophy, Dunnigan type	2348	Primary lipodystrophy	'E881'
Familial steroid-resistant nephrotic syndrome with adrenal insufficiency	506334	Rare adrenal disease	'E8889'
Prepubertal anorexia nervosa	525738	Rare hypothalamic or pituitary disease	'F5000'
Progressive encephalomyelitis with rigidity and myoclonus	438266	Rare diabetes mellitus	'G0481''G0489'
Hypomyelination -hypogonadotropic hypogonadism -hypodontia syndrome	88637	Rare hypothalamic or pituitary disease	'G1110'
Ataxia -telangiectasia	100	Rare endocrine growth disease	'G113'
Spastic paraplegia -intellectual disability-nystagmus -obesity syndrome	521390	Genetic obesity	'G114'
Cerebellar ataxia -hypogonadism syndrome	1173	Rare hypothalamic or pituitary disease	'G118'
Classic stiff person syndrome	443192	Rare diabetes mellitus	'G2582'
Severe neurodegenerative syndrome with lipodystrophy	363400	Primary lipodystrophy	'G3189'
Proximal myotonic myopathy	606	Rare disorder with hypergonadotropic hypogonadism	'G7119'
Myopathy and diabetes mellitus	2596	Rare diabetes mellitus	'G713'
Sudden infant death-dysgenesis of the testes syndrome	168593	Disorder of sex development	'G908'
Kearns -Sayre syndrome	480	Rare parathyroid disease and phosphocalcic metabolism anomaly	'H49811''H49812''H49813''H49819''H49881'
Deafness -hypogonadism syndrome	90646	Rare disorder with hypergonadotropic hypogonadism	'H906'
Satoyoshi syndrome	3130	Rare endocrine growth disease	'M358'
Oncogenic osteomalacia	352540	Rare parathyroid disease and phosphocalcic metabolism anomaly	'M838'
Denys -Drash syndrome	220	Disorder of sex development	'N041'
Lipoprotein glomerulopathy	329481	Rare dyslipidemia	'N078'
HNF1B -related autosomal dominant tubulointerstitial kidney disease	93111	Rare diabetes mellitus	'N118'
Dent disease type 1	93622	Rare parathyroid disease and phosphocalcic metabolism anomaly	'N2581'
Permanent neonatal diabetes mellitus-pancreatic and cerebellar agenesis syndrome	65288	Rare diabetes mellitus	'P702'
Fetal iodine syndrome	1910	Rare thyroid disease	'P722'
Alobar holoprosencephaly	93925	Rare hypothalamic or pituitary disease	'Q042'
X-linked lissencephaly with abnormal genitalia	452	Disorder of sex development	'Q043'
Septo -optic dysplasia spectrum	3157	Rare hypothalamic or pituitary disease	'Q044'
Intellectual disability -obesity -brain malformations -facial dysmorphism syndrome	352530	Genetic obesity	'Q048'
Axenfeld -Rieger syndrome	782	Rare hypothalamic or pituitary disease	'Q1381'
Kallmann syndrome -heart disease syndrome	2326	Rare hypothalamic or pituitary disease	'Q248'
Müllerian aplasia and hyperandrogenism	247768	Disorder of sex development	'Q51818'
Testicular regression syndrome	983	Disorder of sex development	'Q550'
Penile agenesis	49	Disorder of sex development	'Q555'
Persistent Müllerian duct syndrome	2856	Disorder of sex development	'Q558'
46,XX ovotesticular disorder of sex development	2138	Disorder of sex development	'Q560'
46,XY disorder of sex development -adrenal insufficiency due to CYP11A1 deficiency	168558	Disorder of sex development	'Q561'
46,XX disorder of sex development induced by endogenous maternal -derived androgen	325093	Disorder of sex development	'Q562'
Disorder of sex development -intellectual disability syndrome	2983	Disorder of sex development	'Q563'
Acrodysostosis with multiple hormone resistance	280651	Polyendocrinopathy	'Q754'
McCune - Albright syndrome	562	Rare endocrine growth disease	'Q781'
Lipodystrophy -intellectual disability -deafness syndrome	50811	Primary lipodystrophy	'Q788'
Hypohidrotic ectodermal dysplasia -hypothyroidism -ciliary dyskinesia syndrome	1882	Rare thyroid disease	'Q824'
Von Hippel -Lindau disease	892	Rare adrenal disease	'Q858'
Hyposmia -nasal and ocular hypoplasia -hypogonadotropic hypogonadism syndrome	2250	Rare hypothalamic or pituitary disease	'Q870'
Prader -Willi syndrome due to paternal deletion of 15q11q13 type 1	177901	Rare hypothalamic or pituitary disease	'Q871''Q8711''Q8719'
Rubinstein -Taybi syndrome due to CREBBP mutations	353277	Genetic obesity	'Q872'
MOMO syndrome	2563	Genetic obesity	'Q873'
Woodhouse - Sakati syndrome	3464	Rare hypothalamic or pituitary disease	'Q875'
Jung syndrome	2321	Rare hypothalamic or pituitary disease	'Q8781''Q8782''Q8789'
Familial thyroglossal duct cyst	93953	Rare thyroid disease	'Q892'
6q16 microdeletion syndrome	171829	Rare hypothalamic or pituitary disease	'Q935''Q9351''Q9359'
Turner syndrome	881	Rare endocrine growth disease	'Q960'
Turner syndrome due to structural X chromosome anomalies	99413	Rare endocrine growth disease	'Q961'
Mosaic monosomy X	99228	Rare endocrine growth disease	'Q963'
46,XX ovarian dysgenesis -short stature syndrome	444048	Disorder of sex development	'Q968'
Monosomy X	99226	Rare endocrine growth disease	'Q969'
Trisomy X	3375	Rare endocrine growth disease	'Q970'
Tetrasomy X	9	Rare endocrine growth disease	'Q971'
48,XXXY syndrome	96263	Disorder of sex development	'Q981'
45,X/46,XY mixed gonadal dysgenesis	1772	Disorder of sex development	'Q987'
48,XXYY syndrome	10	Disorder of sex development	'Q988'
Tetragametic chimerism	199310	Disorder of sex development	'Q990'
46,XX gonadal dysgenesis	243	Disorder of sex development	'Q991'
Fragile X syndrome	908	Genetic obesity	'Q992'
Xp21 deletion syndrome	261476	Rare adrenal disease	'Q998'
Familial multinodular goiter	276399	Rare thyroid disease	'E042'
Pediatric -onset Graves disease	525731	Rare thyroid disease	'E0500''E0501'
Arachnoid cyst	2356	Rare hypothalamic or pituitary disease	'G930'
Autoimmune enteropathy and endocrinopathy -susceptibility to chronic infections syndrome	391487	Polyendocrinopathy	'K639'
Primary microcephaly -epilepsy -permanent neonatal diabetes syndrome	306558	Rare diabetes mellitus	'Q02'
